# The prevalence, nature and severity of oropharyngeal dysphagia in the acute post-operative phase following curative resection for esophageal cancer

**DOI:** 10.1093/dote/doaf054

**Published:** 2025-07-11

**Authors:** Michelle Hayes, Anna Gillman, Jessie A Elliott, Claire L Donohoe, John V Reynolds, Julie Regan

**Affiliations:** Department of Clinical Speech and Language Studies, Trinity College Dublin, Dublin, Ireland; Department of Speech and Language Therapy, St. James’ Hospital, Dublin, Ireland; Department of Clinical Speech and Language Studies, Trinity College Dublin, Dublin, Ireland; Department of Surgery, St. James’ Hospital, Dublin, Ireland; Department of Surgery, St. James’ Hospital, Dublin, Ireland; Department of Surgery, St. James’ Hospital, Dublin, Ireland; Department of Surgery, Trinity Translational Medicine Institute, Trinity College Dublin, Dublin, Ireland; Department of Clinical Speech and Language Studies, Trinity College Dublin, Dublin, Ireland

**Keywords:** aspiration, curative esophageal cancer, esophagectomy, swallowing, videofluoroscopy

## Abstract

**Background:**

Oropharyngeal dysphagia and aspiration in the early post-esophagectomy period is rarely studied. This study investigated its prevalence, nature and severity, differences across surgical subgroups, and predictors of risk.

**Methods:**

A prospective cohort study was conducted (January 2022–January 2024) at the National Esophageal Cancer Centre. Data was collected on post-operative day (POD) 4 or 5. Swallowing evaluations included videofluoroscopy [Dynamic Imaging Grade of Swallowing Toxicity v2(DIGESTv2), Modified Barium Swallow Impairment Profile (MBSImP), Penetration-Aspiration Scale (PAS)]. Functional Oral Intake Scale (FOIS) was used to identify oral intake status.

**Results:**

N = 30 (25 males) were recruited, mean age (range) of 65 (46-80y), n = 13 2-stage, n = 8 3-stage, and n = 9 transhiatal resections. At POD 4/5, 60% (18/30) showed signs of aspiration, with no differences across surgical groups (*P* = 0.114). Dysphagia per the DIGESTv2 was present in 83% (25/30) of patients, with severe dysphagia in 23% (7/30). MBSImP assessment revealed reduced tongue base retraction (82%), pharyngeal residue (100%) and impaired neo-esophageal clearance (100%). Predictors of aspiration were: pre-operative abnormal FOIS (score < 7) (OR = 7.00, 95%CI 1.2–38.4; *P* = 0.024), and > 65 years (OR = 7.80, 95%CI 1.47–41.6; *P* = 0.016). Predictors for oropharyngeal dysphagia were: abnormal pre-operative FOIS (score < 7) (OR = 7.42, 95%CI 1.22–45.45; *P* = 0.029); age > 65 years (OR = 11.00, 95%CI 1.99–58.8; *P* = 0.006) and neoadjuvant treatment (OR = 7.20, 95%CI 1.08–47.96, *P* = 0.041).

**Conclusion:**

Oropharyngeal dysphagia and aspiration are prevalent in the early period after esophageal cancer surgery. These data should inform an increased input from speech and language specialists in the assessment and management of post-operative patients, and overall caution in the implementation and progression of early *per orum* intake.

## INTRODUCTION

Esophageal Cancer (EC) is the seventh most common cancer worldwide, and the sixth leading cause of cancer-related deaths.^1^^,^^2^ Surgery for EC is complex and associated with a significant risk of post-operative complications and potential long-term morbidity, with a risk of in-hospital mortality of 4.4%.^3–6^ However, due to the centralization of surgery in high volume centres, mortality rates following esophagectomy has markedly decreased in recent years.^7^ Post-operative morbidity is common, at ⁓60%, with post-operative pulmonary complications (PPCs) being the most prevalent risk, with a spectrum of problems including pneumonia, acute lung injury, acute respiratory distress syndrome (ARDS), pleural effusions and aspiration.^8^^,^^9^

In this context, the impact of physiological normalization of swallowing control, and associated airway safety from gross and micro-aspiration, has rarely been studied systematically. This is an important question at a time when enhanced recovery after surgery (ERAS) protocols are increasingly standardized, which typically includes the earliest re-introduction of oral fluids and nutrition as is deemed clinically safe, usually at ⁓48 hours.^10^^,^^11^ In some clinically evident cases, in particular where recurrent laryngeal nerve injury is evident, extreme caution would be required due to the risk of aspiration.^12^ However, more subtle nuanced changes may occur, impact on swallow and aspiration risk, including hyoid displacement, geniohyoid muscle impairment and decreased swallowing pressures, with pharyngeal residue identified as a critical factor in the development of post-operative pneumonia.^13–15^ Other factors, including a prolonged operation time (>6 hours), age and sarcopenia are risk factors for dysphagia and aspiration.^16–18^

Few studies have assessed oropharyngeal dysphagia and aspiration in the early post-operative period. The goal of this study was to understand the physiological and clinical impact of standard operations (2-stage, 3-stage and transhiatal esophagectomy) on swallow function and aspiration in the early period, at a time when early oral intake is being increasingly encouraged in standard clinical practice. We report herein data that may inform clinical practice and approaches within enhanced recovery protocols and increase the role of detailed swallow and aspiration risk assessment.

## METHODS

### Study design

A prospective cohort study was conducted in a single acute hospital setting, over a 24-month period January 2022 to January 2024. The Strengthening the Reporting of Observational Studies in Epidemiology (STROBE) guidelines^19^ were followed (see Appendix A). Ethical approval was granted from the St. James’s Hospital/Tallaght University Hospital Joint Research Ethics Committee Project, ID 0029. The primary author created a Patient and Public Involvement (PPI) group, which included esophageal cancer survivors (>5 years post-surgery) to review the research study design and assisted with the selection of appropriate assessments and outcome measures.

### Participant recruitment

Participants were recruited at the National Esophageal Centre (NEC) in Ireland. All patients had curative intent treatment for esophageal cancer involving surgery. Eligible participants were posted a patient information leaflet, consent form and cover letter, followed by a phone call 7 days later. Informed written and verbal consent were obtained prior to surgery. The study aimed to recruit 60 participants. Based on previous literature in this cancer cohort to estimate an effect size 0.5 at a significance level of 0.05 and a power of 0.8. This sample estimate is consistent with other publications in this area.^20^

### Eligibility criteria

Adults (>18 year) who were deemed appropriate for curative treatment were invited to participate in this research. A diagnosis of esophageal cancer was confirmed by biopsy and treated with curative intent involving surgery alone, or combined with neoadjuvant/adjuvant chemotherapy, +/− radiation. Exclusion criteria included metastatic disease or a history of head and neck cancer, or pre-existing neurological conditions.

All participants were treated with either trimodality therapy (CROSS regimen),^21^ perioperative chemotherapy (FLOT/FOLFOX),^22^^,^^23^ or surgery alone, based on existing guidelines and MDT discussion. Surgical resection was performed ⁓6-weeks post completion of neoadjuvant therapy. Surgery included open transthoracic (two-or three-stage *en bloc*) or a transhiatal resection, combined with a 2-field lymphadenectomy for 2 and 3 stage resections, with a thoracic lymphadenectomy including 4R and 4 L station nodes, and abdominal (D2) dissection in all cases.

As per the Enhanced Recovery After Surgery (ERAS) pathway at the NEC, patients post esophageal resection commence a specific oral intake protocol which typically commenced on post-operative day (POD) 4 or 5 (see Appendix B).

### Study protocol

Swallowing-related outcome measures were collected at the acute phase, POD 4 or 5 depending on surgical approach. Each participant underwent a standardized videofluoroscopy (VFS) and pre-operative oral intake status as rated on the functional oral intake scale (FOIS) was completed.^24^ The first author reviewed the hospitals electronic patient record system to review patient information including risk factors, pre-operative information and post-operative complications experienced.

### VFS protocol

A standardized VFS protocol was completed for all participants (see appendix C). The protocol was designed in conjunction with the surgical consultants. If gross aspiration was observed, the protocol was discontinued for safety purposes. VFS was completed by the first author (MH; an experienced speech and language therapist) and a radiographer. VFS was recorded on TIMS DICOM (Foresight Imaging LCC) using continuous fluoroscopy capturing 25 frames per second as per European recommendations.^25^ Consistencies were prepared according to the International Dysphagia Diet Standardization Initiative (IDDSI) framework using 20% weight to volume w/v) concentration of barium contrast (Maxibar TM 98.45% w/v).^26^^,^^27^ The contrast media was changed to water soluble contrast (Niopam 61.2% w/v Iopamidol equivalent to 300 mg iodine/ml)^28^ given the early post-operative setting. The participants were initially seated upright and trialed with the following consistencies including IDDSI level 0/thin drinks presented by cup/syringe (4 × 5 mls, 3 × 10 mls, 3 × cup sips), IDDSI level 4/puree (3 × 5mls). The participants were then assisted with standing for an anterior–posterior position, identifying the inferior pharynx superiorly and remnants of the cervical esophagus and given IDDSI level 0/thin drinks (3 × 5 mls) where the radiographer tracked the bolus inferiorly toward the gastric cardia/body (if present and not surgically removed). No further trials of dietary consistencies could be trialed due to the ERAS pathway and time needed for neo-esophageal healing.

### Clinical outcomes and swallowing measures

Clinical and surgical data was obtained from the electronic hospital records, these included clinical (age, neoadjuvant therapy and pre-operative FOIS) and surgical (site of anastomosis and oxygen therapy) data were collected to determine if they could predict post-operative aspiration and oropharyngeal dysphagia at the acute post-operative phase. The FOIS identified the oral intake status (tube reliance or food modification/avoidance) of each participant pre- and post-operatively. It is a 7-point ordinal scale where 1–3 indicates a feeding tube requirement, 4–6 modified/avoidance of food/fluid consistencies and 7 no food/fluid restriction.

### VFS data analysis

Three validated tools were used to analyze swallow safety, swallow efficiency and swallow physiology based on VFS studies.

#### Penetration-aspiration scale (PAS)

The PAS is an 8-point ordinal scale and widely recognized tool that describes the degree to which the bolus enters the airway and whether it is subsequently ejected.^29^ Score 1: no airway penetration or aspiration. Scores 2–3: Indicate penetration without aspiration. Scores 4–8: Indicate varying degrees of aspiration, with score 8 representing silent aspiration (no response to the material entering the airway. Aspiration was defined as a rating score of 6–8 across bolus volumes and consistencies, whereas participants who did not experience aspiration were defined as those who were rated as 1–5. Participants were categorized into two groups, participants presenting with (A) Aspiration (PAS 6–8) and (B) No Aspiration (PAS 1–5).

#### The dynamic imaging grade of swallowing toxicity v2

The DIGESTv2 is a validated tool used to determine pharyngeal dysphagia and designed to assess and grade the efficiency and safety of swallowing. DIGESTv2 emphasizes the grading of swallowing toxicity, considering the frequency and severity of unsafe swallowing events.^30^ The presence of pharyngeal dysphagia on the DIGESTv2 protocol; an ordinal scale where 0 = no pharyngeal dysphagia, 1 = mild dysphagia, 2 = moderate dysphagia, 3 = severe dysphagia and 4 = profound pharyngeal dysphagia. The tool integrates both quantitative measures (e.g. amount of residue, penetration, aspiration) and qualitative judgments about the impact of these events on overall swallowing safety. The presence of pharyngeal dysphagia was defined as a rating of grade 1 or more, those who rated 0 were categorized as not having dysphagia.

#### Modified barium swallow impairment profile

Finally, the MBSImP is a validated, standardized tool which evaluates 17 distinct physiologic components of swallowing, categorized into three functional domains: oral (components 1–6), pharyngeal (components 7–16) and esophageal (component 17).^31^ Each component is scored on an ordinal scale where higher scores indicate greater impairment. The tool offers three methods for scoring: (i) Overall Impression (OI), captures the highest impairment observed across swallowing tasks. (ii) Composite Scores, combines scores within the oral and pharyngeal domains. (iii) Swallow-By-Swallow (SS) provides detailed scoring for each swallow across different consistencies and tasks. The validated VFS tools collectively offer a comprehensive approach to assessing and managing swallowing impairment, each focusing on different aspects of swallowing physiology, and the FOIS measuring tube reliance and food modification or avoidance. To evaluate inter-rater reliability of VFS data, a separate author independently re-rated 25% of VFS studies (PAS, MSBImP and DIGESTv2 tools) using a two-way random model assessment on SPSS.

### Statistical analysis

Descriptive statistics were used to illustrate participant characteristics and reported as means with standard deviations (SD) and median with interquartile range (IQR). Subgroup analysis examined median differences in aspiration, dysphagia and pre-existing food avoidance/modification (PAS, DIGESTv2, MSBImP and FOIS) across the three surgical groups. To investigate a difference in swallow safety (PAS), overall pharyngeal dysphagia (DIGESTv2) and swallow physiology (MBSImP) was completed. To examine differences in swallowing across the 3 surgical approaches, a mean rank Kruskal-Wallis H-test was utilized as the non-parametric equivalent of one-way ANOVA comparing means in swallow data across subgroups. To determine if clinical and surgical factors predicted aspiration status (aspirator versus non-aspirator) and oropharyngeal dysphagia (dysphagic versus non-dysphagic), a binary logistic regression analysis was conducted. Based on the number of participants, five independent variables (surgical anastomosis, age (>65 years), pre-existing food avoidance/modification (FOIS), oxygen support, neoadjuvant therapy) were selected based on previous literature and on a visual review of the data. Statistical analysis was carried out using SPSS 27 (SPSS, Inc, Chicago, IL, USA). For all statistical tests, a two-sided α of ˂0.05 was considered statistically significant.

## RESULTS

### Acute phase recruitment

A total of 64 participants (55 males/9 females) were screened via consecutive sampling. 34 participants were withdrawn due to metastatic disease (n = 3), history of head and neck cancer (n = 1), history of progressive neurological disease (n = 3), open-close surgical case due to extensive disease (n = 2), cardiac arrest (n = 1), immediate re-intubation post-operatively (n = 1), acute delirium (n = 1), anastomotic leak (n = 2), aspirated during water-soluble swallow study (n = 2), VFS machine malfunction (n = 1), no radiology report following water-soluble swallow study to proceed with VFS (n = 2), with the highest number of participants (n = 15) were lost due to post-operative pulmonary complications (PPC’s) and deemed too high a risk to transfer out of the intensive care unit. Thirty participants (25 males/5 females) were transferred from the intensive care unit (ICU) or specialist surgical ward and completed the VFS. The mean time since surgery was 4.5 days. A total of 16 (53%) participants were over the age of 65 years, 14 (47%) were under 65 years. Median length of ICU stay was 5.0 days. Median length of hospital stay was 12.5 days. Data on post-operative pulmonary complications, pneumonia and recurrent laryngeal nerve palsy across operative type is highlighted in Table 1: Patient Demographics. The VFS protocol was not completed for 11 (37%) of participants, one participant asked to discontinue the test due to early satiety (VFS study was immediately after the water-soluble test, analyses of the surgical anastomosis) and 10 (33%) participants could not complete the VFS due to gross aspiration and safety concerns.

Inter-rater agreement on VFS rating was good-to-excellent: 81% (ICC 2,2 = 0.814) on MBSImP (98% of responses were within one point difference or less), and 94% and 95% on PAS and DIGESTv2 scores, respectively. The median scores for dysphagia, aspiration and swallow physiology are: DIGESTv2: 1(1–3), PAS 2.3(2–3), MSBImP Total Oral 11 (8–14), MSBImP Total Pharyngeal 11(9.0–17.0) and FOIS 1(1.0–1.0). Table 2 provides descriptive results (mean, SD, median, IQR and range).

### Acute phase aspiration

Participants were placed into 2 groups, those who had experienced (A) Aspiration, or (B) No Aspiration. 18 (60%) participants presented with aspiration (6–8 on PAS) and 12 (40%) had no evidence of aspiration on VFS imaging (1–5 on PAS). 4 (13.3%) participants did not experience airway penetration or aspiration, 1 (3.3%) participant had uncleared penetration, 7 (23.3%) participants effectively cleared penetration, and 18 (60%) participants had uncleared aspiration. The cohort was further divided into three surgical subgroups: 2-stage, 3-stage and transhiatal. In the 2-stage group, 6 (46%) participants experienced aspiration, while 7 (54%) participants had no aspiration. In the 3-stage group, 4 (50%) participants showed evidence of aspiration, while 4 (50%) had no aspiration. In contrast, the transhiatal group had a higher proportion of participants experiencing aspiration, with 8 (89%) exhibiting aspiration and only 1(11%) had no aspiration (see appendix D). There was no statistically significant difference in participants experiencing aspiration across surgical subgroups (*P* = 0.114). No statistically significance difference (*P* = 0.183) was found in those aspirating across anastomotic location (thoracic versus cervical). Table 3 highlights participants presenting with (A) Aspiration and (B) No Aspiration across surgical subgroups.

**Table 1:** Demographics of total participants recruited.

**Recruited**	N = 30	**Surgery Type**		
		**Thoracic Group (**43%)	**Cervical Group** (57%)	
		**2- stage** 13 (43%)	**3-stage** 8 (27%)	**Transhiatal** 9 (30%)
**Age** (mean, range)	64.9 (46–80)	60.5 (46–77)	64.2 (55–76)	71.5 (62–80)
**Sex** Males Females	25 (83%) 5 (17%)	12 1	6 2	7 2
**Cancer Type** Adenocarcinoma Squamous Cell High grade dysplasia	24 (80%) 5 (17%) 1 (3%)	13 0 0	3 5 0	8 0 1
**Cancer Location** Middle Lower Junctional	8 (27%) 10 (33%) 12 (40%)	0 5 8	7 1 0	1 4 4
**Cancer Classification: Pathological Stage** T1 tumors T2 tumors T3 tumors	14 5 11	4 T1N0M0 2 T1N1M0 2 T2N0M0 1 T2N3M0 2 T3N0M0 2 T3N2M0	3 T1N0M0 1 T1N1M0 1 T2N0M0 1 T3N0M0 1 T3N1M0 1 T3N3M0	2 T1N0M0 2 T1N1M0 1 T2N1M0 2 T3N0M0 1 T3N2M0 1 T3N3M0
**ASA (American Society of Anaesthesiologists) Grade** Normal Mild systemic disease Severe systemic disease	3 18 9	3 7 3	0 4 4	0 7 2
**Clavien-Dindo Classification** 19 (64%) respiratory tract infection 7 (23%) Pneumonia 2 (7%) RNL PALSY (Diagnosed by Otolaryngologist**)**	**Grade I (10)**	4	3	3
	**Grade II (4)**	4 Acute delirium Delayed gastric emptying Respiratory infection/lower respiratory tract infection (LRTI)	0	0
	**Grade IIIa (7)**	3 Acute delirium Cardiac arrhythmia/failure Respiratory complications/pulmonary embolism/LRTI Cardiac complications	1 Acute delirium Raised C-reactive protein (CRP)/Consolidation/LRTI	3 Acute delirium Pulmonary complications/chest tube drainage Respiratory complications/consolidation/LRTI/raised CRP Pneumonia
	**Grade IIIb (5)**	1 Pleural effusion Chyle leak	1 Respiratory complications/LRTI	3 Para conduit hernia repair Pneumonia Chyle leak
	**Grade Iva (1)**		1 Recurrent laryngeal nerve injury Pneumonia	
	**Grade IVb (1)**		1 Recurrent laryngeal nerve injury Pneumonia	
	**Grade V (1)**	1 Pneumonia Small bowel obstruction		
	**Grade V (1)**		1 Myocardial infarct	
**Neo-adjuvant treatment** Nil Chemotherapy Chemoradiation	6 10 14	1 6 6	0 2 6	5 2 2
**Wide bore (NGT) for surgical drainage insitu at time of VFS** **Wide Bore NGT removed at time of VFS**	25 (15 aspirated) 5 (3 aspirated)	9 (4 aspirated) 4 (2 aspirated)	7 (3 aspirated) 1 (1 aspirated)	9 (8 aspirated) 0 (0 aspirated)
**In-hospital Follow-up** **Repeat in-hospital VFS**	29 12	13 3	7 (1 RIP) 3	9 6

NGT, nasogastric tube; VFS, videofluoroscopy.

**Table 2:** Acute phase aspiration, pharyngeal dysphagia and oral intake status results

**POD 4/5 (N = 30)**	**Mean +/− Std Dev scores**	**Median/IQR**	**Range**
**PAS**	2.26 +/− 0.73	2.3/2–3	1–8
**DIGESTv2**	1 +/− 1.45	1.0/1–3	0–4
**MBSImP Total Oral**	11 +/−3.72	11/8.0–14.0	1–13
**MBSImP Total Pharyngeal**	13 +/− 4.74	11/9.0–17.0	1–13
**FOIS**	1.0 +/− 0.025	1.0/1.0–1.0	1–7

**Table 3:** Participants presenting with aspiration versus no aspiration across surgical subgroups

**Acute Phase: (N = 30**) **VFS PAS Results**					
**A) Aspiration: 18**			**B) No Aspiration: 12**		
**Surgical Type**					
**Thoracic Anastomosis** (43%)		**Cervical Anastomosis** (57%)			
**2-Stage** **13 (**43%)		**3-Stage** **8 (**27%)		Transhiatal **9** (30%)	
**Aspiration**	**No Aspiration**	**Aspiration**	**No Aspiration**	**Aspiration**	**No Aspiration**
6 PAS 6–8 (46%)	7 PAS 1–5 (54%)	4 PAS 6–8 (50%)	4 PAS 1–5 (50%)	8 PAS 6–8 (89%)	1 PAS 1–5 (11%)

### Acute phase oropharyngeal dysphagia

**Fig. 1:**
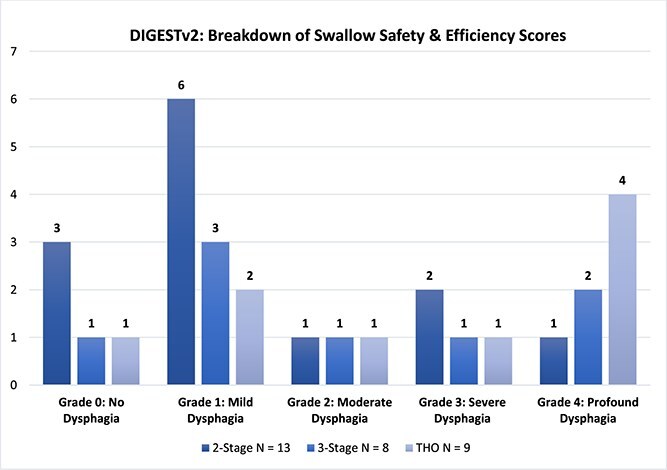
Acute Phase Dynamic Imaging Grade of Swallowing Toxicity v2 Results.

At time of recommencing oral intake, 5 (17%) participants presented with no dysphagia on the DIGESTv2, 11 (37%) had a mild dysphagia, 3 (10%) had moderate dysphagia, 4 (13%) had severe dysphagia, and 7 (23%) had profound dysphagia in swallowing safety and efficiency. 25 (83%) participants presented with grade 1 or more and categorized with dysphagia and 5 (17%) rated 0 therefore categorized as not having dysphagia. Examining dysphagia across surgical subgroups, the majority of participants across all subgroups exhibited a mild impairment in swallowing safety and efficiency. The highest number of participants (3) with no pharyngeal impairment/grade 0 was the 2-stage surgical approach, the highest number of participants (4) with a profound impairment/grade 4 was those who had undergone a transhiatal esophagectomy. The protocol was not completed for 10 (33%) participants due to gross silent aspiration (PAS = 8) and severe or profound impairment (grade = 3 or 4) across all 3 surgical subgroups: 3 (10%) 2-stage, 3 (10%) 3-stage and 4 (13%) transhiatal. There was no statistically significant difference across the three surgical subgroups presenting with dysphagia (*P* = 0.258). Furthermore, there was no statistically significant difference (*P* = 0.133) in those presenting with dysphagia across surgical approaches (thoracic versus cervical anastomosis). See Figure  1 for a breakdown of swallow safety and efficiency scores.

### Acute phase swallow physiology

**Fig. 2:**
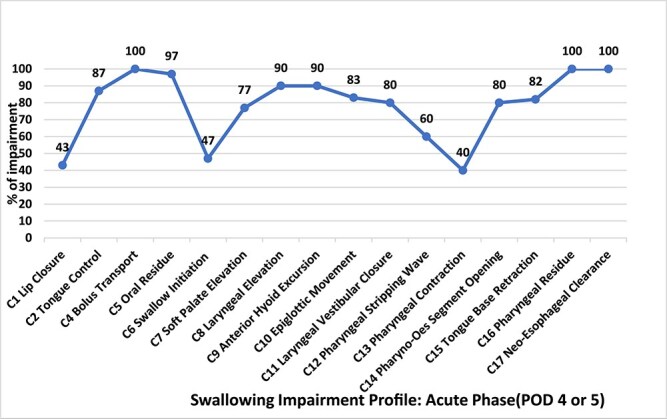
MBSImP Impairment Profile Results.

All participants presented with multiple impairments on the MBSImP with the highest impairment in bolus transfer (100%), oral residue (96%), reduced laryngeal elevation (90%), reduced anterior hyoid excursion (90%), reduced tongue base retraction (82%), pharyngeal residue (100%) and neo-esophageal clearance (82%). See Figure  2 for percentage of participants presenting with oral, pharyngeal and esophageal impairment as per the MBSImP. The components whose median [interquartile range (IQR)] results were impaired (>0) during oral swallow included tongue control 3(1,2), bolus transport/lingual motion 2(2,2) and oral residue 2(1.25,3). The components whose median (IQR) results were impaired (>0) during pharyngeal swallow included soft palate 1(1,1), laryngeal elevation 1(1,1), anterior hyoid excursion 1(1,1), epiglottic movement 1(1,2), laryngeal vestibular closure 1(1,1), pharyngeal stripping wave 1(0,1), pharyngoesophageal segment opening 1.5(1,2), tongue base retraction 2(2,2.75) and pharyngeal residue 2(2,3). The median (IQR) results for the esophageal swallow where impairment was present (>0), included neo-esophageal clearance 1 (1,2).

### Clinical and surgical variables independently associated with aspiration & dysphagia

Participants who presented with pre-existing esophageal dysphagia before surgery, denoted by an abnormal FOIS (score < 7) were associated with an independent increased risk of aspiration-based PAS results at the acute phase (OR 7.00; 95% CI 1.20–38.4; *P* = 0.024). Participants >65 years were determined to be a predictor of aspiration (OR 7.80; 95% CI 1.47–41.6; *P* = 0.016). The remaining variables (neo-adjuvant therapy, site of anastomosis and oxygen therapy) were not found to be predictors of aspiration. Independent predictors of oropharyngeal dysphagia as rated on the DIGESTv2 were abnormal FOIS (OR 7.42; 95% CI 1.22–45.0; *P* = 0.029), > 65 years (OR 11.00, 95% CI 1.99–58.8; *P* = 0.006) and neoadjuvant therapy (OR 7.2; 95% CI 1.08–47.9; *P* = 0.041. The remaining variables (site of anastomosis and oxygen therapy) were not found to be predictors of oropharyngeal dysphagia. See Table 4 (Univariate regression analysis) for predictors of aspiration and oropharyngeal dysphagia.

**Table 4:** Acute phase predictors of aspiration & oropharyngeal dysphagia: univariant binary regression analysis

**Predictors of Aspiration**	**Odds ratio**	**95% CI**	***P*-value**
Pre-operative FOIS <7	6.99	1.20–37.9	0.024
Age > 65	7.80	1.47–41.2	0.016
Site of Anastomosis	–	–	0.181
Neoadjuvant therapy (Chemotherapy & Radiation)	–	–	0.237
O_2_ therapy when commencing oral intake	–	–	0.051
**Predictors of Oropharyngeal Dysphagia**			
Pre-operative FOIS <7	7.42	1.22–45.0	0.029
Age >65	11.00	1.99–60.5	0.006
Site of Anastomosis	–	–	0.072
Neoadjuvant therapy (Chemotherapy & Radiation)	7.2	1.08–47.9	0.041
O_2_ therapy when commencing oral intake	–	–	0.260

## DISCUSSION

This observational study explored the prevalence, nature and severity of oropharyngeal dysphagia in the immediate post-operative period. The findings demonstrate a high incidence of acute post-operative oropharyngeal dysphagia, in 83% of participants, with 60% aspirating using these defined and validated measures. There were no statistically significant differences in aspiration rates across surgical subgroups (2-stage, 3-stage or transhiatal), or surgical approaches (thoracic versus cervical anastomosis). Perhaps unexpectedly, in the 2-stage resection cohort, with no neck dissection or anastomosis, 46% (6) of patients had signs of aspiration at this time point, with 10% (3) of participants’ VFS assessments discontinued due to airway safety concerns. In the cervical anastomosis group, 50% of the 3-stage participants were aspirating at this phase, and the highest number of aspiration rates 89%, was seen in the transhiatal group. 10% (3) of the 3-stage participants and 13% (4) of the transhiatal group had the VFS discontinued due to potential respiratory concerns following gross aspiration. There was no statistically significant difference in those presenting with oropharyngeal dysphagia across surgical subgroups (2-stage, 3-stage or transhiatal), or surgical approaches (thoracic versus cervical anastomosis). However, impairment in swallowing safety and efficiency was evident across all surgical subgroups. 10% (3) of participants presented with a grade 2/moderate dysphagia, 13% (4) had a grade 3/severe dysphagia and 23% (7) had grade 4/profound dysphagia. Moreover, impaired swallow pathophysiology was evident in the immediate post-operative phase with all participants presenting with multiple impairments on the MBSImP. Aspiration rates represented the patients who were recovering well following resection who could travel to radiology for assessment. It is likely that aspiration rates are underestimated in this study, as patients with co-morbidities and those experiencing complications following surgery were excluded from the research. Furthermore, the study protocol had to be discontinued for one third of the participants, which may have also led to an underestimated aspiration rate.

Impaired airway protection and oropharyngeal dysphagia may be affected by a variety of factors in the acute post-operative period. Age >65 years was independently significant for aspiration in this study, consistent with previous reports.^32^ Sarcopenia is a known independent risk factor for dysphagia, with loss of muscle mass and strength to swallow safely, impacting the ability to chew nutritious food potentially leading to malnutrition and dehydration.^30^^,^^31^ Furthermore, it could be hypothesized that some participants may have experienced a presbyphagia prior to undergoing multimodal treatment and therefore presented with an oropharyngeal dysphagia post-resection. Another notable finding of the study was that 20 (67%) of participants reported they had pre-operative dysphagia before surgery and an abnormal FOIS (score < 7), and this cohort was at increased risk of aspiration. This may support the hypothesis that in participants with an abnormal FOIS may have had suboptimal nutrition and may have been at higher risk of clinically relevant sarcopenia. The intricate inter-relationship between the motor pattern of pharyngeal swallow into the neo-esophagus and aspiration, is an important theme.^33^ High-flow oxygen is standard in the first few post-operative days, but it may impact the synchronized breathing-swallowing pattern.^34^ The safe synchronized breathing-swallowing pattern can be negatively impacted by pre-existing and acquired pulmonary disease.^35^ Other factors may include patient fatigue, pain, infection and medications. Overall, the findings highlight the variability in swallowing impairment severity across the three different subgroups. These results indicate that a considerable proportion of participants at the acute phase are at an increased risk of aspirating including those who had undergone a 2-stage resection but particularly those who had undergone a transhiatal esophagectomy. Predictors of aspiration and oropharyngeal dysphagia that we report may inform post-operative management. Impaired airway protection and swallowing dysfunction may lead to an increased risk of pneumonia, hospital costs, length of stay and readmission to hospital post-resection. Establishing a framework of care such as early identification and dysphagia management incorporating swallow screening tools and modified diets could improve patient outcomes and post-operative complications. Early identification leading to timely intervention including both clinical assessment and swallow rehabilitation, may improve patient outcomes and be more cost-effective for the hospital.

We acknowledge some limitations. Firstly, a high percentage of participants failed the screening during the recruitment process which resulted in a smaller sample size being recruited than initially planned, which may have implications for the power of the subgroup analysis. Recruitment was likely impacted by the timing of the VFS, however the researchers wanted to determine participants at risk of aspiration and oropharyngeal dysphagia when typically recommencing oral intake following resection. Due to the challenges with recruitment, it was difficult to make comparisons between surgical subgroups. Non the less the authors were keen to explore swallowing difficulties in both cervical and transthoracic surgical approaches to guide future research. While there was limited representation across surgical subgroups, the researchers were keen to evaluate dysphagia by including the transthoracic group as this has not been investigated in previous studies. In addition, it was unknown if the participants had a pre-existing oropharyngeal dysphagia prior to resection. Future research would benefit from investigating these parameters at diagnosis and following neoadjuvant treatment. Furthermore, a number of participants presented with a wide bore nasogastric tube (NGT) at the time of VFS, the association between wide bore NGT and aspiration should be investigated in future studies. The strengths of the study are that it is the first prospective observational research using consecutive sampling examining the prevalence, nature and severity of oropharyngeal dysphagia in the immediate acute post-operative setting including both transthoracic and transhiatal approaches. Moreover, the original findings should inform further research, and also the safe progression of oral intake in the post-esophagectomy period.

In conclusion, the key finding from this novel data is that impaired airway protection is evident across surgical subtypes in the acute phase when patients are re-commencing oral intake after 4 or 5 post-operative days. This suggests that early swallow and aspiration screening should be considered as part of the team approach within ERAS to optimize perioperative care and improve outcomes. At this time, this is rarely done. We encourage a standardized protocol for assessing and managing oropharyngeal dysphagia, and suggest potential clinical benefits. This study suggests an expanded role for the speech and language therapist within the esophageal cancer multidisciplinary team with the need for the development of a framework of care with a tailored protocol including pre-operative assessment, post-operative swallow screening, dysphagia rehabilitation and structured clinical pathway for this patient group. These studies throughout the esophageal cancer treatment pathway, from diagnosis through survivorship, should be pursued in collaborative research protocols to better defined the physiologic disturbance of surgery, and the mechanisms and short- and long-term consequences. Finally, with an increasing use of minimally invasive and robotic assisted minimally invasive esophagectomy (RAMIE) internationally, these data may provide a metric for comparative analysis.

## Notes

Specific author contributions: Michelle Hayes (Conceptualization, Data curation, Formal analysis, Methodology, Project administration, Resources, Visualization, Writing — original draft, Writing—review & editing), Anna Gillman (Methodology, Writing—review & editing), Jessie A. Elliott (Methodology, Writing—review & editing), Claire L. Donohoe (Formal analysis, Writing—review & editing), John V. Reynolds (Conceptualization, Methodology, Supervision, Writing—review & editing), Julie Regan (Conceptualization, Methodology, Supervision, Writing—review & editing).

Financial support: Funding was provided through the School of Linguistics, Speech and Communication Sciences, Trinity College Dublin PhD Scholarship Award.
