# Correction to: The prevalence, nature and severity of oropharyngeal dysphagia in the acute post-operative phase following curative resection for esophageal cancer

**DOI:** 10.1093/dote/doaf074

**Published:** 2025-09-02

This is a correction to: Michelle Hayes, Anna Gillman, Jessie A Elliott, Claire L Donohoe, John V Reynolds, Julie Regan, The prevalence, nature and severity of oropharyngeal dysphagia in the acute post-operative phase following curative resection for esophageal cancer, *Diseases of the Esophagus*, Volume 38, Issue 4, August 2025, https://doi.org/10.1093/dote/doaf054

The following changes have been made to the originally published manuscript.

In section *SUMMARY*, the last sentence is emended to read: “This data should inform […]” instead of: “These data should inform […]”.

In section **INTRODUCTION**, the last sentence of the second paragraph is emended to read: “[…] for dysphagia and aspiration.”” instead of: “[…] for dysphagia and aspiration risk.”

Under heading **CFS data analysis**, text in the first sentence is emended to read: “Three validated tools were used to analyze […]” instead of: “Three validated tools were used to analyse […]”.

Under subheading Penetration Aspiration Scale (PAS), the first sentence is emended to read: “The PAS is an 8-point ordinal scale […]” instead of: “The penetration aspiration scale (PAS) is an 8-point ordinal scale […]”.

Subheading “The dynamic imaging grade of swallowing toxicity v2” is emended to read: “The Dynamic Imaging Grade of Swallowing Toxicityv2 (DIGESTv2)”. The first sentence of the first paragraph is emended to read: “The DIGEST v2 is a validated tool […]” instead of: “The dynamic imaging grade of swallowing toxicity (DIGEST) v2 is a validated tool […]”.

Subheading “Modified barium swallow impairment profile” is emended to read: “Modified Barium Swallow Impairment Profile (MBSImP)”. The first sentence of the first paragraph is emended to read: “Finally, the MBSImP is a validated, standardized tool […]” instead of: “Finally, the modified barium swallow impairment profile (MBSImP) is a validated, standardized tool […]”.

Under subheading **Penetration Aspiration Scale (PAS)**, the first sentence is emended to read: “The PAS is an 8-point ordinal scale […]” instead of: “The penetration aspiration scale (PAS) is an 8-point ordinal scale […]”.

Text in the first column of Table 2 is emended to read: “MBSImP” instead of: “MSBImP”.

Below Table 2, text is deleted: “POD, post-operative day; IQR, interquartile range; DIGESTv2, Dynamic Imaging Grade of Swallowing Toxicity v2; MBSImP, MBS Impairment Profile; FOIS, Functional Oral Intake Scale.”

Below Table 3, text is deleted: “VFS, videofluoroscopy.”

Text at the top of the first paragraph, second column, page 6: “[…] retraction (100%), […]” instead of: : “[…] retraction (82%), […]”.

Text at the end of the first paragraph, second column, page 6: “[…] included neo-esophageal clearance […]” instead of: “[…] included esophageal clearance […]”.

Fig. 2 legend is changed to read: “MBSImP Impairment Profile Results” instead of: “MBS Impairment Profile Results”.

Below Table 4, text is deleted: “FO18 Functional Oral Intake Scale”.

Text in the second column, second paragraph, page 8: “Recruitment was likely impacted […]” instead of: “Recruited was likely impacted […]”.

Text in the first column, second paragraph, page 9: “[…] the key finding from this novel data” instead of: “[…] the key finding from these novel data”.

The emendations have been made to the article.

